# Spatial Profiling Identifies Prognostic Features of Response to Adjuvant Therapy in Triple Negative Breast Cancer (TNBC)

**DOI:** 10.3389/fonc.2021.798296

**Published:** 2022-01-10

**Authors:** Arutha Kulasinghe, James Monkman, Esha T. Shah, Nicholas Matigian, Mark N. Adams, Ken O’Byrne

**Affiliations:** ^1^ University of Queensland Diamantina Institute, The University of Queensland, Brisbane, QLD, Australia; ^2^ School of Biomedical Sciences, Centre for Genomics and Personalised Health, Queensland University of Technology, Brisbane, QLD, Australia; ^3^ QFAB Bioinformatics, The University of Queensland, Brisbane, QLD, Australia; ^4^ Princess Alexandra Hospital, Brisbane, QLD, Australia

**Keywords:** TNBC (Triple negative breast cancer), spatial proteomics, adjuvant chemotherapy, tumor microenvirnonment, spatial transcriptomics

## Abstract

Triple-negative breast cancer (TNBC) is an aggressive subtype of breast cancer that has few effective treatment options due to its lack of targetable hormone receptors. Whilst the degree of tumour infiltrating lymphocytes (TILs) has been shown to associate with therapy response and prognosis, deeper characterization of the molecular diversity that may mediate chemotherapeutic response is lacking. Here we applied targeted proteomic analysis of both chemotherapy sensitive and resistant TNBC tissue samples by the Nanostring GeoMx Digital Spatial Platform (DSP). By quantifying 68 targets in the tumour and tumour microenvironment (TME) compartments and performing differential expression analysis between responsive and non-responsive tumours, we show that increased ER-alpha expression and decreased 4-1BB and MART1 within the stromal compartments is associated with adjuvant chemotherapy response. Similarly, higher expression of GZMA, STING and fibronectin and lower levels of CD80 were associated with response within tumour compartments. Univariate overall-survival (OS) analysis of stromal proteins supported these findings, with ER-alpha expression (HR=0.19, p=0.0012) associated with better OS while MART1 expression (HR=2.3, p=0.035) was indicative of poorer OS. Proteins within tumour compartments consistent with longer OS included PD-L1 (HR=0.53, p=0.023), FOXP3 (HR=0.5, p=0.026), GITR (HR=0.51, p=0.036), SMA (HR=0.59, p=0.043), while EPCAM (HR=1.7, p=0.045), and CD95 (HR=4.9, p=0.046) expression were associated with shorter OS. Our data provides early insights into the levels of these markers in the TNBC tumour microenvironment, and their association with chemotherapeutic response and patient survival.

## Introduction

Breast cancer (BC) is the most commonly diagnosed form of cancer and is the leading cause of cancer related mortality in women worldwide (1). The molecular subtypes of BC have distinct pathological features and are characterised by amplification of their hormone receptors, estrogen receptor (ER), progesterone receptor (PR) and human epidermal growth factor 2 (HER2), or the absence thereof in triple-negative BC (TNBC) (2, 3). Treatment strategies for ER/PR positive and HER2-positive breast cancer include hormone targeted therapies like tamoxifen (4, 5), aromatase inhibitors (6) and HER-2 specific monoclonal antibody, trastuzumab (7) which are often used in combination with chemotherapeutic agents like paclitaxel (8).

TNBC, however, which accounts for 15% of diagnosed breast cancers, lacks these key targets, and as such remains difficult to treat with current generations of therapies (9). Standard treatment for TNBC include anthracycline, taxane and platinum-based regimens (2). In patients with advanced TNBC and chemotherapy resistant tumours, PARP inhibition and immunotherapy have been gaining traction (10), while patients with metastatic TNBC with *BRCA1/2* deficiency, can respond well to PARP inhibitors including Olaparib and Talazoparib (11, 12).

Methods to stratify TNBC patients are currently lacking, however elevated levels of tumour infiltrating lymphocytes (TILs) have been shown to indicate favourable response to chemotherapy, particularly for TNBC and HER-2 positive patients (13, 14). TNBC tumours are considered to be the most immunogenic BC subtype, and display expression of programmed cell death ligand 1 (PD-L1) on both tumour and immune cells, and increased TIL infiltration (15, 16). TNBC tumours also display a higher burden for non-synonymous mutations that are capable of inducing T-cell anti-tumour responses (17). Anti PD-1 antibody pembrolizumab has received accelerated approval from the FDA for PD-L1 positive TNBC patients in combination with chemotherapy (18). Clinical trials with existing immunotherapies in combination with approved chemotherapies and targeted therapies, like PARP inhibitors, and those investigating novel immunotherapy agents are currently in progress (16).

To garner insight into the properties of TNBC that may benefit therapy stratification, a greater understanding of the TIL repertoire within the tumour microenvironment is necessary. The cellular composition of, and the interactions that occur within this niche between cancer cells and the host immune components are likely to influence disease progression. In this study we have used the Nanostring GeoMX Digital Spatial Profiler (DSP) to gain tumour and stroma compartment specific protein expression profiles of TNBC tumours. Our data indicates the utility of such multiplex approaches to discover the properties of primary tumours that may lead to their propensity to progress despite treatment, thereby distinguishing those patients most likely to respond.

## Materials and Methods

This study has Queensland University of Technology Human Research Ethics Committee Approval (UHREC #2000000494). A tissue microarray (TMA) containing twenty-four triple negative breast cancer specimens with clinicopathological findings was obtained in collaboration with Tristar Technology Group (USA). The cohort was comprised of patients with histological grade 3 invasive ductal carcinoma who received first-line FEC (5-fluoruracil, epirubicin and cyclophosphamide) adjuvant chemotherapy. Patients were defined by complete response (CR), where no evidence of disease was found during subsequent follow up, while progressive disease (PD) patients were refractory to treatment and subsequently succumbed to disease within the follow-up period.

### Nanostring GeoMX Digital Spatial Profiling (DSP)

The TNBC TMA slides were stained and analysed on the Nanostring GeoMx^®^ Digital Spatial Profiling (DSP) platform by the Systems Biology and Data Science Group at Griffith University (Gold Coast, Australia). Visualisation markers consisted of Pan-cytokeratin and CD45. Protein panel consisted of 68 antibodies including human immune cell core panel, immuno-oncology (IO) drug target, immune activation, immune cell typing, pan-tumour, cell death, and PI3K/AKT panels. Slides were processed as per manufacturer’s instructions and tumour/stroma demarcated by masking on PanCK^+^ or PanCK^-^ regions, respectively. Antibody barcodes were counted on Ncounter^®^ platform as per manufacturer’s instructions and External RNA Controls Consortium (ERCC) QC performed in DSP analysis suite prior to outputting data for bioinformatic analysis.

### Bioinformatic Analysis

Data analysis was performed by Queensland Cyber Infrastructure Foundation (QCIF, Qld, Australia). Data was evaluated by principal component analysis and coefficients of variation were assessed to determine suitability of the RUV-III normalisation method (19, 20). Differential analysis was performed within Limma packages (21) and sparse partial least squares-discriminant analysis (sPLS-DA) was performed within mixOmics package (22). The Kaplan-Meier survival analysis and Cox proportional hazards models was constructed within R studio (23) using Survival package (24) and plots generated by ggplot2 (25).

## Results

### TNBC Cohort

**Figure 1:**
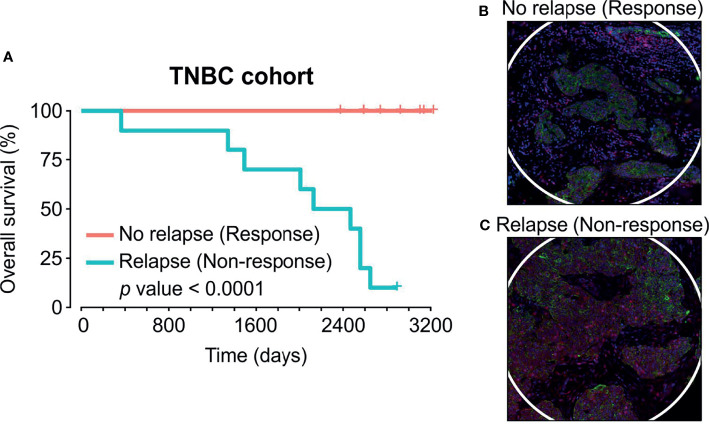
Adjuvant chemotherapy cohort and regions captured by Nanostring GeoMX Digital Spatial Profiling protein assay. **(A)** Kaplan-Meier survival curve of TNBC cohort which consisted of 15 patients who responded to chemotherapy (red line) and 9 patients who relapsed and succumbed to disease within follow-up time (blue line). **(B, C)** Spatial profiling was performed on PanCK+ (Tumour) and PanCK- (Stroma) areas. Green = PanCK, Red=CD45.

To identify proteins that influence disease progression in response to chemotherapy, we evaluated a cohort of TNBC tissue samples from women undergoing adjuvant chemotherapy in collaboration with TriStar Technology Group (USA). Our annotated tissue microarray (TMA) with 8.5-year follow-up data included 15 cases without relapse (responders) following therapy versus 9 cases with tumour relapse (non-responders) (**Table 1**). All non-responsive patients subsequently succumbed to metastatic disease following surgery within the follow-up period time, while responders remained alive (**Figure 1A**). One non-responsive patient with progressive disease was censored at final follow up time point.

**Table 1:** Clinicopathological details of the TNBC patient cohort.

	Responders (n=15)	Non-responders (n=9)
**Age**	53 (34,77)	55 (28,76)
**Grade**	3	3
**T Stage**		
** 1**	8	5
** 2**	7	3
** 3**	–	–
** 4**	–	1
**Nodal Met**		
** 1**	15	7
** 2**	–	2
**Survival (Days)**	2747 (2374,3226)	1989 (365,2892)
**Metastasis (Liver, Lung)**	0	9

*Data are mean with range in brackets.

### Identification of Differentially Expressed Proteins by the Nanostring GeoMX DSP Assay

**Figure 2:**
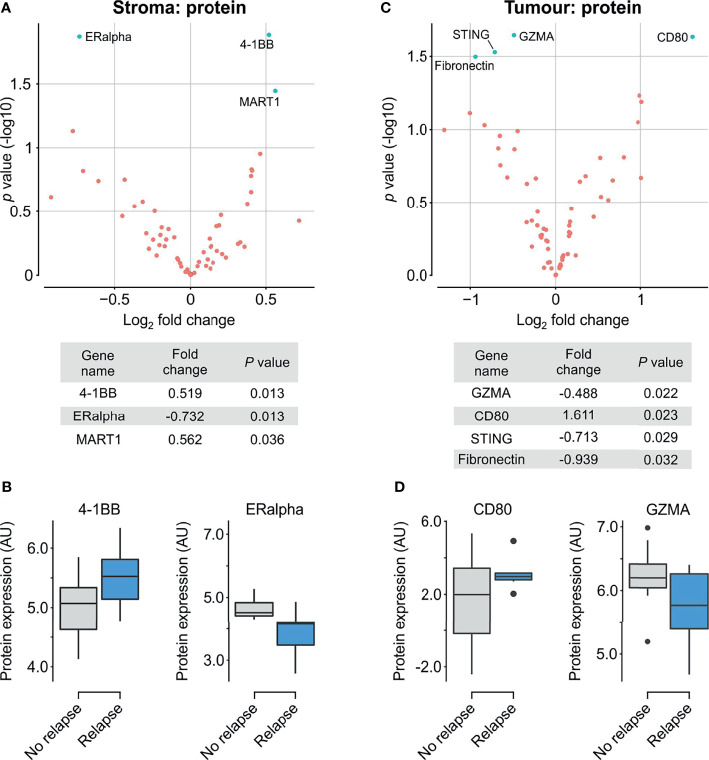
Differential protein expression comparing relapse and complete response. **(A)** *Upper panel*. Volcano scatter plot showing stromal enrichment of log2 fold change in proteins from responders (left half) vs non-responders (right half) ranked by significance (-log_10_ *P* value). *Lower panel.* List of top three significant deregulated proteins ranked by *P* value. **(B)** Boxplots indicating enrichment of 41BB and ERα in non-responders and responders, respectively. **(C)** *Upper panel.* Volcano scatter plot showing tumour region enrichment of log2 fold change in proteins from responders (left half) vs non-responders (right half) ranked by significance (-log_10_ *P* value). *Lower panel.* List of top four significant deregulated proteins ranked by *P* value. **(D)** Boxplots indicating enrichment of CD80 and GZMA in non-responders and responders, respectively.

We compared the two groups of patient chemotherapy responders and non-responders by performing multiplex proteomics using the Nanostring GeoMx^®^ Digital Spatial Profiling (DSP) platform. We identified key deregulated proteins within the tumour and stromal compartments independently by masking on PanCK+/PanCK- regions, respectively. (**Figures 1B, C**). Analysis of the PanCK- stroma within each core indicated higher expression of estrogen receptor alpha (ERα) in responsive patients, with concurrent decreased levels 41BB and MART1 (**Figures 2A, B**). Differential analysis of tumour compartments indicated the enrichment of GZMA, STING and fibronectin in responsive patients, while CD80 levels were higher within refractory tumours (**Figures 2C, D**).

### Prognostic Benefit of Proteins Between Responder/Non-Responder Patient Groups

**Figure 3:**
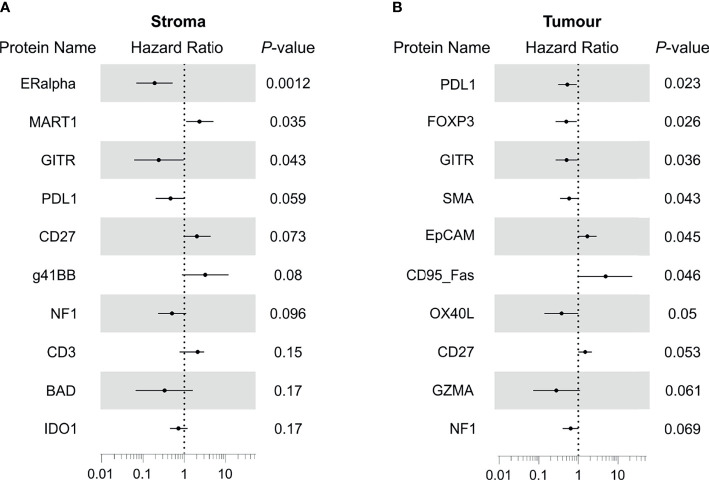
Identification of proteins with prognostic associations for treatment of TNBC with adjuvant chemotherapy. **(A)** Forest plot showing hazard ratio and 95% confidence interval for stromal proteins that possessed overall survival associations. **(B)** Forest plot showing hazard ratio and 95% confidence interval for tumour proteins that possessed overall survival associations. HR>1 indicates association with poorer outcome.

We next sought to investigate the associations between our protein expression data and overall survival (OS). Cox regression indicated DE protein ERα (HR=0.19, p=0.0012) and GITR (HR=0.24, p=0.043) within the stroma to be associated with better prognosis, while DE protein MART1 (HR=2.3, p=0.035) indicated poorer outcome (**Figure 3A**). Interestingly, stromal PD-L1 (HR=0.46, p=0.059) expression demonstrated a trend for improved outcome.

Notably, evaluation of tumour compartments showed little consistency with DE results, with the expression of PD-L1 (HR=0.53, p=0.023), FOXP3 (HR=0.5, p=0.026), GITR (HR=0.51, p=0.036), SMA (HR=0.59, p=0.043) implicated in improved outcome. Conversely, tumour EPCAM (HR=1.7, p=0.045), and CD95 (HR=4.9, p=0.046) were associated with poorer outcome.

**Figure 4:**
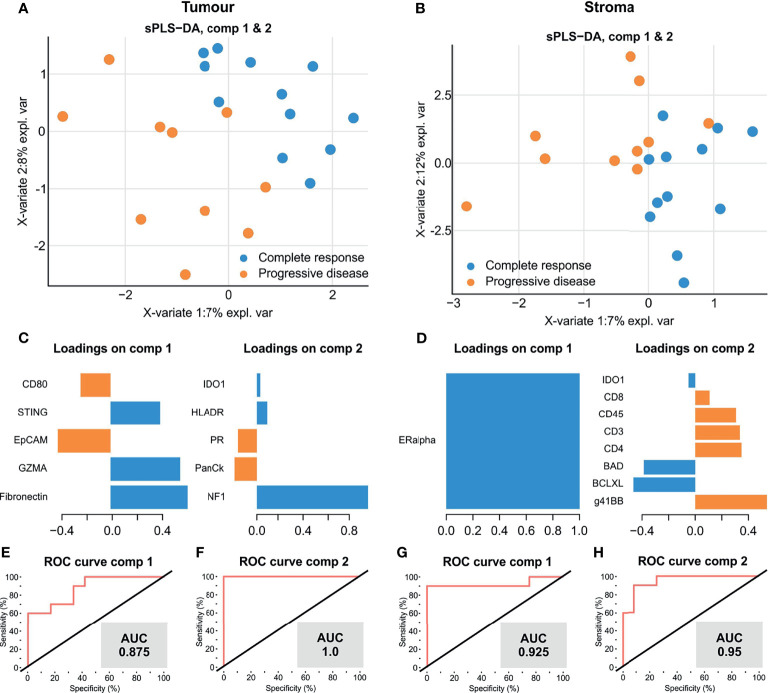
A multi-protein signature discriminates TNBC tumours with complete response to adjuvant chemotherapy. **(A, B)** sPLSDA discrimination of samples by optimal protein signatures in tumour **(A)** and stroma **(B)**. **(C, D)**. Component features of each signature in tumour **(C)** and stroma **(D, E–H)** ROC curve performance of each signature to discriminate patient response groups.

To further investigate factors which influenced disease progression, we utilised a multivariate analysis by sparse partial least-squares discriminant analysis (sPLSDA) to identify a minimal signature that distinguishes TNBC therapy response. sPLSDA is a statistical method to identify directionally weighted features that collectively discriminate groups of samples. Signatures within tumour (**Figure 4A**) and stroma (**Figure 4B**) compartments were able to effectively stratify patients by response to therapy. Our tumour compartment specific multivariate signature for response was largely consistent with the DE results (**Figure 4C**), comprising higher levels of fibronectin, GZMA and STING and lower EPCAM and CD80 (AUC = 0.875) (**Figure 4E**). Interestingly, NF1, a negative regulator of the RAS/MAPK pathway almost solely comprised a second tumour signature, indicative of therapy response (AUC=1) (**Figure 4F**). Similarly, a stromal signature composed solely of ERα was indicative of response (**Figure 4D**) (AUC=0.925) (**Figure 4G**). A second multivariate stromal signature composed of T cell costimulatory agent 41BB as well as CD8, CD45, CD3, CD4 with apoptotic regulators BAD and BCLXL also discriminated response (AUC=0.95) (**Figure 4H**). Our discovery data suggest that we have identified a potential unique multi-protein signature to identify those TNBC tumours most likely to respond to adjuvant chemotherapy.

## Discussion

Breast cancer is a heterogenous disease, wherein the TNBC subtype demonstrates a particular refractory response to treatment due to lack of canonical hormone directed targets. TIL infiltration in TNBC has been shown to be a key predictor of response to neoadjuvant chemotherapy (13), in addition to forming the basis for efficacy of current generations of immune checkpoint inhibitors that reinvigorate anti-tumour immune activity. Despite this, little is known regarding relative levels of TIL subsets, and the greater immune composition in TNBC tumours. Thus, an opportunity to investigate these properties and their relationship with clinical outcomes exists. Here we evaluated a preliminary cohort of TNBC tumours from patients that responded to adjuvant chemotherapy and compared them to those that exhibited treatment resistance resulting in progressive disease.

To the best of our knowledge, this is the first study employing proteomic DSP to examine the TNBC tumour microenvironment to discern chemotherapy treatment outcomes. Herein, we extend earlier spatial studies (26) by identifying differentially expressed proteins and protein signatures that associate with prognosis and treatment response. However, our study has a number of limitations. For example, our findings require validation in larger independent retrospective cohorts of TNBC tumours, and in future, trialling within prospectively collected tissues. Moreover, our findings could be further examined in cohorts of neoadjuvant chemotherapy, an established therapy modality in TNBC, and associated with pathological complete response (pCR) (27–29). Future investigations should also utilise orthogonal approaches (immunohistochemistry) to qualify abundance of TIL infiltration and validate experimental findings from the DSP platform (30). Furthermore, biological characterisation and validation of putative targets within immunocompetent murine models is required to delineate their cellular roles in chemotherapeutic response. Nonetheless, our findings putatively identify tumour and stroma compartment-specific proteins that may have prognostic and therapy outcome implications in archival FFPE tissue taken routinely during cancer treatment.

Differential analysis implicated increased stromal ERα and decreased MART1 and 41BB to be associated with response. ERα is typically expressed by luminal breast epithelial cells and forms an axis of hormone directed therapy. Its role within human stromal cells is relatively poorly defined (31), however ERα expression in cancer associated fibroblasts (CAFs) has been observed (32), and its activation has been implicated in the promotion of pro-tumorigenic angiogenesis in breast tumours (33). Its stromal upregulation in responding patients (**Figure 2**) and association with a favourable therapy response (**Figures 3**, **4**) might indicate that such angiogenesis also benefits chemotherapy delivery, thereby improving drug efficacy. 4-1BB, TNF receptor superfamily 9 (TNFSR9) is responsible for effector CD8 T cell function and survival, and its suppression in responding patients in our data suggests some moderation of T cell activity (34). Consistently, our findings identified the association of stromal GITR expression with favourable therapy response (**Figure 3**). Activation of this TNFR superfamily member is co-stimulatory for T cell proliferation and anti-apoptotic cell survival (35, 36). Our findings suggest that expression of these factors, at least within the stroma, participate in subtle modulation of T cell activity to impact TNBC therapy response.

Our study additionally identified that within the tumour compartments, responding patients exhibited higher levels of GMZA, STING and fibronectin and reduced levels of CD80 (**Figure 3**). Furthermore, our analysis demonstrated that each of these proteins contributed to a multi-protein signature discerning between chemotherapy responsive and nonresponsive tumours (**Figure 4**). Of these proteins, granzyme A (GZMA) is a key cytolytic protease for cytotoxic activity of CD8+ T cells and NK cells (37), while STING acts as a cytosolic DNA sensor that induces production of interferon α/β. Accordingly, recent clinical evaluation of the DNA damage Immune Response (DDIR) Signature, which is representative of cGAS-STING activation (38), demonstrated that in chemotherapy treated TNBC, DDIR positivity associated with improved overall survival and disease free survival (39). Taken together, our findings are consistent with increased immune cell recruitment and activation, promoting immuno-reactive functions that potentially synergise with chemotherapy, resulting in enhanced response.

Further to our proteomics analysis of tumour compartment proteins, it is worth noting that the survival-associated proteins appeared independent of those proteins found through our differential analysis. This is unlike the proteins enriched within the stomal compartment of patient responders that were largely associated with longer OS. Within the tumour compartment, those tumours with increased levels of PD-L1, FOXP3 and GITR expression were associated with longer OS (**Figure 3**). These findings point to a potential beneficial role for T-regs and infiltration of GITR expressing TILs in modulating immune activity for patient survival. Similarly, our observation that tumour cell PD-L1 expression in TNBC is positively associated with improved OS is consistent with published findings (15, 40). Furthermore, PD-L1 positive disease displayed improved OS in response to anti-PD-L1 immunotherapy in phase III IMpassion130 trial (41), and has subsequently shown improved outcome in response to combination therapies (18).

In addition to our study, other deep profiling methods to evaluate TIL subtypes and cellular markers associated with therapy response in TNBC have included multiplex imaging (42), bioinformatic deconvolution of bulk public mRNA data (43, 44), and RNAseq of tumours stratified by CD8+ TIL abundance (45, 46). Our data here provides an additional layer of insight into TNBC tumour composition. Future investigations may benefit from larger TNBC cohorts stratified by TIL abundance to further delineate immune cell roles in therapy response.

In summary, we have shown the utility for spatially mapping the tumour microenvironment in TNBC to identify putative markers associated with response and resistance to adjuvant chemotherapy. Whilst preliminary, the data supports further investigation and validation of the identified biomarkers which can be of prognostic utility for TNBC. The integration of spatial proteomic datasets, using approaches as described in this manuscript, are highly novel and like to lead to the identification of novel biomarkers for TNBC.

## Data Availability Statement

The raw data supporting the conclusions of this article will be made available by the authors, without undue reservation.

## Ethics Statement

The studies involving human participants were reviewed and approved by Queensland University of Technology Human Research Ethics Committee Approval (UHREC #2000000494). The patients/participants provided their written informed consent to participate in this study.

## Author Contributions

Design/concept: AK and KO’B. Experimental: AK, JM, and MA. Analysis: all authors. Writing and review: all authors. All authors contributed to the article and approved the submitted version.

## Funding

This work is supported by project grants and fellowships for AK from NHMRC (1157741), Cure Cancer (1182179), the PA Research Foundation (KOB), and an International Association for the Study of Lung Cancer Foundation (IASLC) Foundation Award (MNA).

## Conflict of Interest

NM is employed by QCIF Bioinformatics.

The remaining authors declare that the research was conducted in the absence of any commercial or financial relationships that could be construed as a potential conflict of interest.

## Publisher’s Note

All claims expressed in this article are solely those of the authors and do not necessarily represent those of their affiliated organizations, or those of the publisher, the editors and the reviewers. Any product that may be evaluated in this article, or claim that may be made by its manufacturer, is not guaranteed or endorsed by the publisher.

## References

[1] Sung H Ferlay J Siegel RL Laversanne M Soerjomataram I Jemal A. Global Cancer Statistics 2020: GLOBOCAN Estimates of Incidence and Mortality Worldwide for 36 Cancers in 185 Countries. CA: A Cancer J Clin (2021) 71:209–49. doi: 10.3322/caac.21660 33538338

[2] Harbeck N Gnant M. Breast Cancer. Lancet (2017) 389:1134–50. doi: 10.1016/S0140-6736(16)31891-8 27865536

[3] Fragomeni SM Sciallis A Jeruss JS. Molecular Subtypes and Local-Regional Control of Breast Cancer. Surg Oncol Clin North Am (2018) 27:95–120. doi: 10.1016/j.soc.2017.08.005 PMC5715810 29132568

[4] Dowsett M Houghton J Iden C Salter J Farndon J A'hern R. Benefit From Adjuvant Tamoxifen Therapy in Primary Breast Cancer Patients According Oestrogen Receptor, Progesterone Receptor, EGF Receptor and HER2 Status. Ann Oncol (2006) 17:818–26. doi: 10.1093/annonc/mdl016 16497822

[5] Davies C Godwin J Gray R Clarke M Cutter D Darby S. Relevance of Breast Cancer Hormone Receptors and Other Factors to the Efficacy of Adjuvant Tamoxifen: Patient-Level Meta-Analysis of Randomised Trials. Lancet (2011) 378:771–84. doi: 10.1016/S0140-6736(11)60993-8 PMC3163848 21802721

[6] Dowsett M Cuzick J Ingle J Coates A Forbes J Bliss J. Meta-Analysis of Breast Cancer Outcomes in Adjuvant Trials of Aromatase Inhibitors Versus Tamoxifen. J Clin Oncol (2010) 28:509–18. doi: 10.1200/JCO.2009.23.1274 19949017

[7] Cameron D Piccart-Gebhart MJ Gelber RD Procter M Goldhirsch A De Azambuja E. 11 Years' Follow-Up of Trastuzumab After Adjuvant Chemotherapy in HER2-Positive Early Breast Cancer: Final Analysis of the HERceptin Adjuvant (HERA) Trial. Lancet (London England) (2017) 389:1195–205. doi: 10.1016/S0140-6736(16)32616-2 PMC5465633 28215665

[8] Tolaney SM Barry WT Dang CT Yardley DA Moy B Marcom PK. Adjuvant Paclitaxel and Trastuzumab for Node-Negative, HER2-Positive Breast Cancer. N Engl J Med (2015) 372:134–41. doi: 10.1056/NEJMoa1406281 PMC4313867 25564897

[9] Mehanna J Haddad FG Eid R Lambertini M Kourie HR. Triple-Negative Breast Cancer: Current Perspective on the Evolving Therapeutic Landscape. Int J Women's Health (2019) 11:431–7. doi: 10.2147/IJWH.S178349 PMC6682754 31447592

[10] Nagayama A Vidula N Bardia A. Novel Therapies for Metastatic Triple-Negative Breast Cancer: Spotlight on Immunotherapy and Antibody-Drug Conjugates. Oncol (Williston Park) (2021) 35:249–54. doi: 10.3390/jcm9040940 33983696

[11] Keung MY Wu Y Badar F Vadgama JV. Response of Breast Cancer Cells to PARP Inhibitors Is Independent of BRCA Status. J Clin Med (2020) 9:940. doi: 10.3390/jcm9040940 PMC7231148 32235451

[12] Cortesi L Rugo HS Jackisch C. An Overview of PARP Inhibitors for the Treatment of Breast Cancer. Targeted Oncol (2021) 16:255–82. doi: 10.1007/s11523-021-00796-4 PMC8105250 33710534

[13] Denkert C Von Minckwitz G Darb-Esfahani S Lederer B Heppner BI Weber KE. Tumour-Infiltrating Lymphocytes and Prognosis in Different Subtypes of Breast Cancer: A Pooled Analysis of 3771 Patients Treated With Neoadjuvant Therapy. Lancet Oncol (2018) 19:40–50. doi: 10.1016/S1470-2045(17)30904-X 29233559

[14] Ahn S Chung YR Seo AN Kim M Woo JW Park SY. Changes and Prognostic Values of Tumor-Infiltrating Lymphocyte Subsets After Primary Systemic Therapy in Breast Cancer. PLoS One (2020) 15:e0233037. doi: 10.1371/journal.pone.0233037 32401825 PMC7219779

[15] Beckers RK Selinger CI Vilain R Madore J Wilmott JS Harvey K. Programmed Death Ligand 1 Expression in Triple-Negative Breast Cancer Is Associated With Tumour-Infiltrating Lymphocytes and Improved Outcome. Histopathology (2016) 69:25–34. doi: 10.1111/his.12904 26588661

[16] Keenan TE Tolaney SM. Role of Immunotherapy in Triple-Negative Breast Cancer. J Natl Compr Canc Netw (2020) 18:479–89. doi: 10.6004/jnccn.2020.7554 32259782

[17] Narang P Chen M Sharma AA Anderson KS Wilson MA. The Neoepitope Landscape of Breast Cancer: Implications for Immunotherapy. BMC Cancer (2019) 19:200. doi: 10.1186/s12885-019-5402-1 30832597 PMC6399957

[18] Kwapisz D. Pembrolizumab and Atezolizumab in Triple-Negative Breast Cancer. Cancer Immunol Immunother (2021) 70:607–17. doi: 10.1007/s00262-020-02736-z PMC10992894 33015734

[19] Risso D Ngai J Speed TP Dudoit S. Normalization of RNA-Seq Data Using Factor Analysis of Control Genes or Samples. Nat Biotechnol (2014) 32:896–902. doi: 10.1038/nbt.2931 25150836 PMC4404308

[20] Molania R Gagnon-Bartsch JA Dobrovic A Speed TP. A New Normalization for Nanostring Ncounter Gene Expression Data. Nucleic Acids Res (2019) 47:6073–83. doi: 10.1093/nar/gkz433 PMC6614807 31114909

[21] Ritchie ME Phipson B Wu D Hu Y Law CW Shi W. Limma Powers Differential Expression Analyses for RNA-Sequencing and Microarray Studies. Nucleic Acids Res (2015) 43:e47. doi: 10.1093/nar/gkv007 25605792 PMC4402510

[22] Rohart F Gautier B Singh A Lê Cao K-A. Mixomics: An R Package for ‘Omics Feature Selection and Multiple Data Integration. PloS Comput Biol (2017) 13:e1005752. doi: 10.1371/journal.pcbi.1005752 29099853 PMC5687754

[23] Rstudio Team. RStudio: Integrated Development for R. RStudio. Boston, MA (2020). Available at: http://www.rstudio.com/.

[24] Therneau T. A Package for Survival Analysis in R (2021). Available at: https://CRAN.R-project.org/package=survival.

[25] Wickham H. Ggplot2: Elegant Graphics for Data Analysis. New York: Springer-Verlag (2016).

[26] Stewart RL Matynia AP Factor RE Varley KE. Spatially-Resolved Quantification of Proteins in Triple Negative Breast Cancers Reveals Differences in the Immune Microenvironment Associated With Prognosis. Sci Rep (2020) 10:6598–8. doi: 10.1038/s41598-020-63539-x PMC7170957 32313087

[27] Mieog J van der Hage J Van De Velde C. Neoadjuvant Chemotherapy for Operable Breast Cancer. J Br Surg (2007) 94:1189–200. doi: 10.1002/bjs.5894 17701939

[28] Rastogi P Anderson SJ Bear HD Geyer CE Kahlenberg MS Robidoux A. Preoperative Chemotherapy: Updates of National Surgical Adjuvant Breast and Bowel Project Protocols B-18 and B-27. J Clin Oncol (2008) 26:778–85. doi: 10.1200/JCO.2007.15.0235 18258986

[29] Berruti A Amoroso V Gallo F Bertaglia V Simoncini E Pedersini R. Pathologic Complete Response as a Potential Surrogate for the Clinical Outcome in Patients With Breast Cancer After Neoadjuvant Therapy: A Meta-Regression of 29 Randomized Prospective Studies. J Clin Oncol (2014) 32:3883–91. doi: 10.1200/JCO.2014.55.2836 25349292

[30] Monkman J Kim H Mayer A Mehdi A Matigian N Cumberbatch M. IL-2 Stromal Signatures Dissect Immunotherapy Response Groups in Non-Small Cell Lung Cancer (NSCLC). medRxiv (2021) 2021.2008.2005.21261528. doi: 10.1101/2021.08.05.21261528

[31] Rothenberger NJ Somasundaram A Stabile LP. The Role of the Estrogen Pathway in the Tumor Microenvironment. Int J Mol Sci (2018) 19(2):611. doi: 10.3390/ijms19020611 PMC5855833 29463044

[32] Knower KC Chand AL Eriksson N Takagi K Miki Y Sasano H. Distinct Nuclear Receptor Expression in Stroma Adjacent to Breast Tumors. Breast Cancer Res Treat (2013) 142:211–23. doi: 10.1007/s10549-013-2716-6 24122391

[33] Péqueux C Raymond-Letron I Blacher S Boudou F Adlanmerini M Fouque M-J. Stromal Estrogen Receptor-α Promotes Tumor Growth by Normalizing an Increased Angiogenesis. Cancer Res (2012) 72:3010–9. doi: 10.1158/0008-5472.CAN-11-3768 22523036

[34] Bartkowiak T Curran MA. 4-1bb Agonists: Multi-Potent Potentiators of Tumor Immunity. Front Oncol (2015) 5:117. doi: 10.3389/fonc.2015.00117 26106583 PMC4459101

[35] Watts TH. TNF/TNFR Family Members in Costimulation of T Cell Responses. Annu Rev Immunol (2005) 23:23–68. doi: 10.1146/annurev.immunol.23.021704.115839 15771565

[36] Placke T Kopp H-G Salih HR. Glucocorticoid-Induced TNFR-Related (GITR) Protein and Its Ligand in Antitumor Immunity: Functional Role and Therapeutic Modulation. Clin Dev Immunol (2010) 2010:239083. doi: 10.1155/2010/239083 20936139 PMC2948872

[37] Zhou Z He H Wang K Shi X Wang Y Su Y. Granzyme A From Cytotoxic Lymphocytes Cleaves GSDMB to Trigger Pyroptosis in Target Cells. Science (2020) 29(6494):368. doi: 10.1126/science.aaz7548 32299851

[38] Parkes EE Walker SM Taggart LE Mccabe N Knight LA Wilkinson R. Activation of STING-Dependent Innate Immune Signaling by S-Phase-Specific DNA Damage in Breast Cancer. J Natl Cancer Institute (2017) 109:djw199. doi: 10.1093/jnci/djw199 PMC5441301 27707838

[39] Sharma P Barlow WE Godwin AK Parkes EE Knight LA Walker SM. Validation of the DNA Damage Immune Response Signature in Patients With Triple-Negative Breast Cancer From the SWOG 9313c Trial. J Clin Oncol Off J Am Soc Clin Oncol (2019) 37:3484–92. doi: 10.1200/JCO.19.00693 PMC7194448 31657982

[40] Al-Jussani GN Dabbagh TZ Al-Rimawi D Sughayer MA. Expression of PD-L1 Using SP142 CDx in Triple Negative Breast Cancer. Ann Diagn Pathol (2021) 51:151703. doi: 10.1016/j.anndiagpath.2021.151703 33454500

[41] Schmid P Adams S Rugo HS Schneeweiss A Barrios CH Iwata H. Atezolizumab and Nab-Paclitaxel in Advanced Triple-Negative Breast Cancer. N Engl J Med (2018) 379:2108–21. doi: 10.1056/NEJMoa1809615 30345906

[42] Keren L Bosse M Marquez D Angoshtari R Jain S Varma S. A Structured Tumor-Immune Microenvironment in Triple Negative Breast Cancer Revealed by Multiplexed Ion Beam Imaging. Cell (2018) 174:1373–87.e1319. doi: 10.1016/j.cell.2018.08.039 30193111 PMC6132072

[43] Lv Y Lv D Lv X Xing P Zhang J Zhang Y. Immune Cell Infiltration-Based Characterization of Triple-Negative Breast Cancer Predicts Prognosis and Chemotherapy Response Markers. Front Genet (2021) 12:616469. doi: 10.3389/fgene.2021.616469 33815462 PMC8017297

[44] Zhu SY Ma D Shao ZM Yu KD. Prognostic Effect of Microenvironment Phenotype in Triple-Negative Breast Cancer: Biomarker Analysis of a Prospective Trial. Front Mol Biosci (2021) 8:752154. doi: 10.3389/fmolb.2021.752154 34621789 PMC8490613

[45] Gruosso T Gigoux M Manem VSK Bertos N Zuo D Perlitch I. Spatially Distinct Tumor Immune Microenvironments Stratify Triple-Negative Breast Cancers. J Clin Invest (2019) 129:1785–800. doi: 10.1172/JCI96313 PMC6436884 30753167

[46] Hammerl D Martens JWM Timmermans M Smid M Trapman-Jansen AM Foekens R. Spatial Immunophenotypes Predict Response to Anti-PD1 Treatment and Capture Distinct Paths of T Cell Evasion in Triple Negative Breast Cancer. Nat Commun (2021) 12:5668. doi: 10.1038/s41467-021-25962-0 34580291 PMC8476574

